# Vesicular Stomatitis Virus-Based Epstein-Barr Virus Vaccines Elicit Strong Protective Immune Responses

**DOI:** 10.1128/jvi.00336-22

**Published:** 2022-04-11

**Authors:** Xiang-Wei Kong, Xiao Zhang, Guo-Long Bu, Hui-Qin Xu, Yin-Feng Kang, Cong Sun, Qian-Ying Zhu, Run-Bo Ma, Zheng Liu, Yi-Xin Zeng, Mu-Sheng Zeng, Zhu-Long Hu

**Affiliations:** a State Key Laboratory of Oncology in South China, Collaborative Innovation Center for Cancer Medicine, Guangdong Key Laboratory of Nasopharyngeal Carcinoma Diagnosis and Therapy, Sun Yat-sen University Cancer Center (SYSUCC), Guangzhou, China; b Cryo-EM Centre, Southern University of Science and Technology, Shenzhen, Guangdong Province, China; c Zhongshan School of Medicine, Sun Yat-sen University, Guangzhou, Guangdong, China; d Guangdong-Hong Kong Joint Laboratory for RNA Medicine, Guangzhou, China; Lerner Research Institute, Cleveland Clinic

**Keywords:** EBV, prophylactic, VSV, vaccine, gB, gHgL

## Abstract

Epstein-Barr virus (EBV), the first identified human tumor virus, is etiologically associated with various kinds of malignant and benign diseases, accounting for 265,000 cancer incident cases and 164,000 cancer deaths in 2017. EBV prophylactic vaccine development has been gp350 centered for several decades. However, clinical studies show that gp350-centered vaccines fail to prevent EBV infection. Advances in the EBV infection mechanisms shed light on gB and gHgL, the two key components of the infection apparatus. In this study, for the first time, we utilized recombinant vesicular stomatitis virus (VSV) to display EBV gB (VSV-ΔG-gB/gB-G) or gHgL (VSV-ΔG-gHgL). *In vitro* studies confirmed successful virion production and glycoprotein presentation on the virion surface. In mouse models, VSV-ΔG-gB/gB-G or VSV-ΔG-gHgL elicited potent humoral responses. Neutralizing antibodies elicited by VSV-ΔG-gB/gB-G were prone to prevent B cell infection, while those elicited by VSV-ΔG-gHgL were prone to prevent epithelial cell infection. Combinatorial vaccination yields an additive effect. The ratio of endpoint neutralizing antibody titers to the endpoint total IgG titers immunized with VSV-ΔG-gHgL was approximately 1. The ratio of IgG1/IgG2a after VSV-ΔG-gB/gB-G immunization was approximately 1 in a dose-dependent, adjuvant-independent manner. Taken together, VSV-based EBV vaccines can elicit a high ratio of epithelial and B lymphocyte neutralizing antibodies, implying their unique potential as EBV prophylactic vaccine candidates.

**IMPORTANCE** Epstein-Barr virus (EBV), one of the most common human viruses and the first identified human oncogenic virus, accounted for 265,000 cancer incident cases and 164,000 cancer deaths in 2017 as well as millions of nonmalignant disease cases. So far, no prophylactic vaccine is available to prevent EBV infection. In this study, for the first time, we reported the VSV-based EBV vaccines presenting two key components of the EBV infection apparatus, gB and gHgL. We confirmed potent antigen-specific antibody generation; these antibodies prevented EBV from infecting epithelial cells and B cells, and the IgG1/IgG2a ratio indicated balanced humoral-cellular responses. Taken together, we suggest VSV-based EBV vaccines are potent prophylactic candidates for clinical studies and help eradicate numerous EBV-associated malignant and benign diseases.

## INTRODUCTION

Epstein-Barr virus (EBV), also known as human herpesvirus 4 (HHV-4), is a double-stranded DNA virus that was the first identified human tumor virus (1). Since the discovery of EBV in 1964 (2), tremendous effort has been made to develop a prophylactic vaccine to prevent EBV infection and numerous etiologically related malignant and benign diseases, where EBV infection results in 265,000 incident cases and 164,000 deaths of nasopharyngeal carcinoma (NPC), Burkitt’s lymphoma (BL), Hodgkin lymphoma (HL), and gastric cancer (GC) (1, 3–5).

To answer the call to generate prophylactic vaccines, intensive research has concentrated on gp350 (6), which is the first identified ligand for EBV infection into B cells (7) and the most abundant glycoprotein on the surface of EBV virions (8). Additionally, antibodies against gp350 show neutralizing effects (9, 10). Thus, it is reasonable to make gp350 the primary target for vaccine development (11). By incorporating gp350 into different forms of carriers, such as monomers (12), oligomers (13, 14), nanoparticles (15, 16), vaccinia viruses (17), recombinant adenoviruses (18), varicella-zoster viruses (VZVs) (19), and Newcastle disease viruses (NDVs) (20, 21), many gp350-centered vaccines have emerged. To date, however, clinical trials of gp350-centered vaccines have shown no protection against EBV infection (22).

Exciting advances in EBV infection mechanism studies provide new possibilities for identifying targets for vaccine design. To recognize B cells, in addition to gp350 binding to CD21 (7), gp42, which attaches to gHgL to form a triplex, binds to HLA-II (23, 24). For epithelial cells, gHgL recognizes integrins (25), NMHC-IIA (26), and EphA2 (27) to initiate virion-cell binding. To complete virion infection, hypothetically, gB transforms from a prefusion structure to a postfusion structure to mediate virion-cell fusion in both B cells and epithelial cells (28). NRP1 recognition by gB also plays a role in EBV infection of epithelial cells (29). Thus, neutralizing antibodies against gB and gHgL potentially can prevent EBV infection in both B cells and epithelial cells. Attempts have been made to present EBV gB and gHgL in oligomers (30, 31), nanoparticles (32), and VLPs (21). Results suggest the production of high titers of B cell or epithelial cell neutralizing antibodies by vaccination.

Vesicular stomatitis virus (VSV) is a negative-strand single-strain RNA virus. Since its genome cannot be incorporated into the host genome, VSV contains only five nonoverlapping genes, there are no reported deaths from VSV infection, and it is easy to produce, VSV is an ideal platform for antigen presentation (33, 34). To date, numerous VSV-based vaccines against HIV (35), H5N1 (36), Zika virus (37), EV71 (38), etc., are under investigation. Among them, the VSV-based Ebola vaccine, ZEBOV-GP (39), is the first licensed VSV-based vaccines in the United States (40) and Europe (41), which provides strong evidence for the safety and efficacy of VSV-based vaccines. Here, we presented the key glycoproteins of EBV, gB and gHgL, on the surface of VSV. *In vitro* and *in vivo* studies had confirmed high antigenicity, identified neutralizing antibodies, and depicted the unique immune characteristics of both vaccines.

## RESULTS

### Preparation and characterization of VSV-based EBV vaccines.

**FIG 1:**
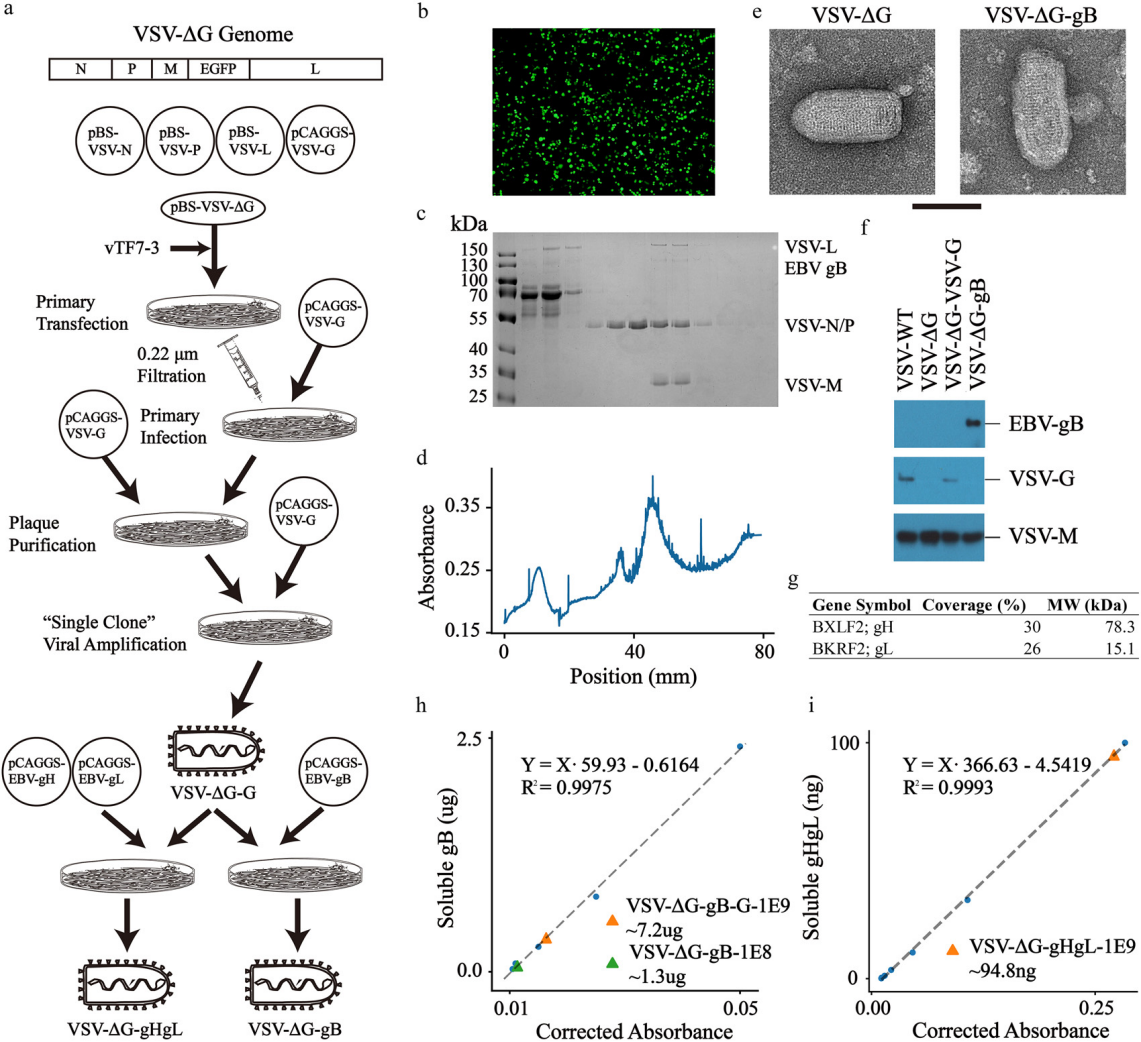
Preparation and characterization of recombinant VSV. (a) The schematic representation of the recombinant VSV production workflow. (b) Fluorescence microscopy images of recombinant VSV. Since the VSV-G gene was replaced by EGFP, the green cells represented virion infection and propagation. (c and d) The outcome of continuous sucrose gradient centrifugation. *A*_280_ was recorded during fractionation. For each fraction, samples were prepared for SDS-PAGE followed by Coomassie brilliant blue staining. Fractions of interest were characterized by the *A*_280_ peak and the simultaneous appearance of VSV-L, VSV-N/P, and VSV-M. (e) Purified virions were bullet-shaped, similar to the wild type, under TEM. The black scale bar represents the length of 100 nm. (f and g) To further confirm protein presentation, immunoblotting and LC-MS were carried out, and target proteins were readily characterized. (h and i) The quantity of proteins of interest on VSV-ΔG assessed by the double-antibody sandwich ELISA. Blue dots stand for corrected absorbance of soluble gB or gHgL at the indicated concentration measured for fitting standard curves.

The workflow of recombinant VSV construction is illustrated in Fig. 1a. Briefly, to construct VSV-ΔG-gB/gHgL, we replaced the VSV-G gene with enhanced green fluorescent protein (EGFP) as the virus amplification indicator. By following a previously reported reversed genetic system (42), we simultaneously transfected VSV-N, VSV-P, VSV-G, VSV-L, and recombinant VSV genomes into host cells. Green fluorescence was observed 48 h after the initial transfection and 24 h after supernatant infection (Fig. 1b). To increase virion homogeneity, we applied plaque purification, and the virions were named VSV-ΔG.

VSV-ΔG presenting EBV gB, EBV gB-G (the intravirion domains were replaced with the corresponding fractions of VSV-G to try enhancing the surface presentation efficiency), and EBV gHgL were collected and purified by continuous sucrose gradient centrifugation. *A*_280_ scanning from the top to the bottom of the centrifuge tube indicated that the location of virions was approximately 50 mm from the top (Fig. 1d). To confirm virion integrity, VSV-ΔG-gB was used as an example. SDS-PAGE showed that all four components of VSV, with the exception of VSV-G, could be observed at the same location; EBV gB could be observed at the corresponding positions (Fig. 1c); and TEM further confirmed intact bullet-shaped virions (Fig. 1e). To confirm the presentation of EBV gB, we applied immunoblotting and showed no VSV-G, but EBV gB presented on VSV-ΔG (Fig. 1f). Due to the lack of immunoblotting antibodies, we could not verify EBV gHgL by immunoblotting. Instead, we successfully verified the presentation of EBV gHgL by liquid chromatography-mass spectrometry (LC-MS) (Fig. 1g).

Due to the lack of receptor binding and fusion-triggering VSV-G, VSV-ΔG could not initiate virion infection. Moreover, EBV gB or EBV gHgL alone could not complete virion infection. Thus, to quantify virions of VSV-ΔG-gB, VSV-ΔG-gB-G, and VSV-ΔG-gHgL, we generated a standard curve of the VSV genome using PFU-known wild-type VSV and calculated the sample titer, called qTiter. To quantify the amount of gB presented, we applied the double-antibody sandwich enzyme-linked immunosorbent assay (ELISA). Approximately 1.3 μg EBV gB or 7.2 μg EBV gB-G was present on the surface of 1E8 qTiter VSV-ΔG-gB or 1E9 qTiter VSV-ΔG-gB-G virions, respectively (Fig. 1h); however, merely 9.48 ng of EBV gHgL was present on the surface of 1E8 qTiter VSV-ΔG-gHgL (Fig. 1i). The difference in presentation efficacy might be attributed to the structural similarity, where VSV-G (43, 44) and gB (28) share a similar triplex structure while gHgL (45) is in monomeric form.

### VSV-ΔG-gB/gB-G elicited strong, characteristic humoral responses.

**FIG 2:**
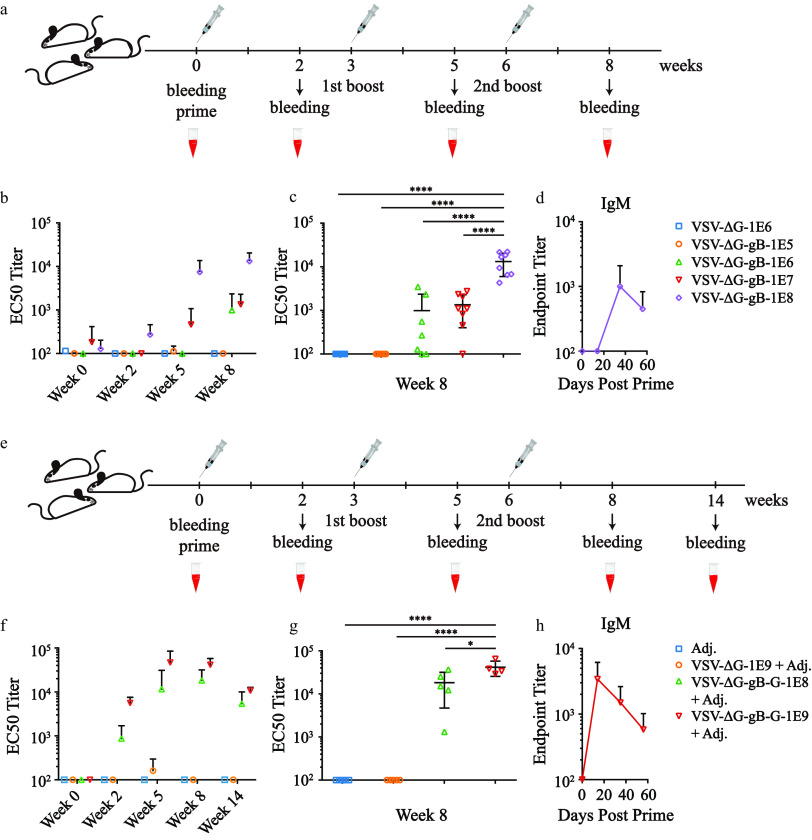
Potent humoral immune responses elicited by VSV-ΔG-gB or VSV-ΔG-gB-G vaccination. (a) The immunization schedule diagram without adjuvant. (b) The kinetics of total IgG EC_50_ titers vaccinated with VSV-ΔG-gB without adjuvant detected by ELISA. Each dot represents the mean titer within each group, and each error bar denotes the standard deviations within each group. *n* = 8 mice per group; one from the group given 1E6 qTiter VSV-ΔG and the other one from the group given 1E6 qTiter VSV-ΔG-gB were dead due to improper bleeding. Other animals were in good status throughout the experiment. (c) To compare dose-dependent effects, we extracted 2nd boost data from panel b and performed one-way analysis of variance (ANOVA) following Tukey’s multiple-comparison test. Each dot represents an individual animal, and horizontal bars represent the mean and standard deviation. (d) IgM titers of the highest-dosage group vaccinated with VSV-ΔG-gB without adjuvant were also measured by ELISA. Each dot represents the mean titer within each group, and each error bar denotes the standard deviation within each group. (e) The immunization schedule diagram with adjuvant. (f) The kinetics of total IgG EC_50_ titers for vaccination with VSV-ΔG-gB-G with aluminum adjuvant detected by ELISA. Each dot represents the mean titer within each group, and each error bar denotes the standard deviation within each group. *n* = 5 mice per group. All animals were in good status throughout the experiment. (g) To compare dose-dependent effects, we extracted 2nd boost data from panel f and performed one-way ANOVA following Tukey’s multiple-comparison test. Each dot represents an individual animal, and horizontal bars represent the means and standard deviations. (h) IgM titers of the highest-dosage group vaccinated with VSV-ΔG-gB-G with aluminum adjuvant were also measured by ELISA. Each dot represents the mean titer within each group, and each error bar denotes the standard deviation within each group. *, *P* < 0.05; **, *P* < 0.01; ***, *P* < 0.001; ****, *P* < 0.0001.

To evaluate the potential use of VSV-ΔG as an EBV vaccine carrier, we carried out *in vivo* experiments with VSV-ΔG-gB and VSV-ΔG-gB-G. The schedule of vaccination with no adjuvant and bleeding is shown in Fig. 2a. Total IgG titers were measured by ELISA (Fig. 2b). Considering that only 1.3 μg of gB was present in 1E8 qTiter VSV-ΔG-gB, the results indicated that as little as 1E6 qTiter VSV-ΔG-gB, corresponding to 13 ng of EBV gB, was enough to induce 1E3 IgG 50% effective concentration (EC_50_) titers, implying potent antigenicity (Fig. 1h and 2b). Compared to lower dosages, higher dosages elicited not only higher titers of IgG but also more homogeneous humoral responses (Fig. 2c). IgM titers reached their peak after the 1st boost (Fig. 2d).

To explore the synergistic effect of adjuvants, we used the most widely applied aluminum adjuvant. With a similar vaccination procedure (Fig. 2e), high titers of IgG could be detected (Fig. 2f). Consistent with the results of vaccination without adjuvant, a higher dosage helped promote not only a higher but also more homogeneous humoral response (Fig. 2g). Fourteen weeks postprime, antibodies against gB dropped but remained readily detectable, implying long-lasting humoral responses (Fig. 2f). IgM titers reached a peak 3 weeks earlier than nonadjuvanted vaccination (Fig. 2d and h).

### VSV-ΔG-gB-G elicited B cell-biased nAbs.

**FIG 3:**
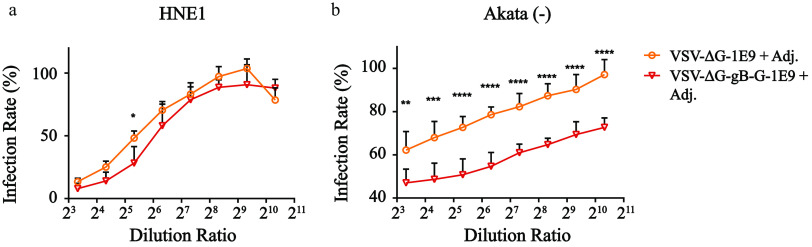
B cell-biased neutralizing antibodies elicited by VSV-ΔG-gB-G vaccination. (a and b) The epithelial cell and the B cell neutralizing kinetics. Infection rates were calculated by dividing the raw infection rates by the mean raw infection rates for three positive controls where no serum was mixed with Akata-EBV prior to EBV infection. Each dot represents the mean normalized infection rate of the indicated dilution ratio, and the error bar denotes the standard deviation. Two-way ANOVA following Sidak’s multiple-comparison test was adopted to perform statistical analysis in panels a and b. *, *P* < 0.05; **, *P* < 0.01; ***, *P* < 0.001; ****, *P* < 0.0001.

EBV utilizes gB to infect both B cells and epithelial cells; therefore, we evaluated whether antibodies elicited by VSV-ΔG-gB-G could protect cells from EBV infection. We prepared Akata-EBV as previously described (27, 29, 46). The inhibition kinetics determined by flow cytometry showed that VSV-ΔG, the negative control, bore some nonspecific neutralizing effects. However, as the dilution ratio continued to increase, significant infection differences could be observed in both B cells and epithelial cells, indicating successful generation of neutralizing antibodies (nAbs). Strikingly, the endpoint titer of epithelial nAbs was far less than that of B cells (40 compared to >1,024) (Fig. 3). Currently, no similar result has been reported using other carriers to present EBV gB (21, 30, 31).

### IgG subtype profile reveals unique immune responses independent of adjuvants.

**FIG 4:**
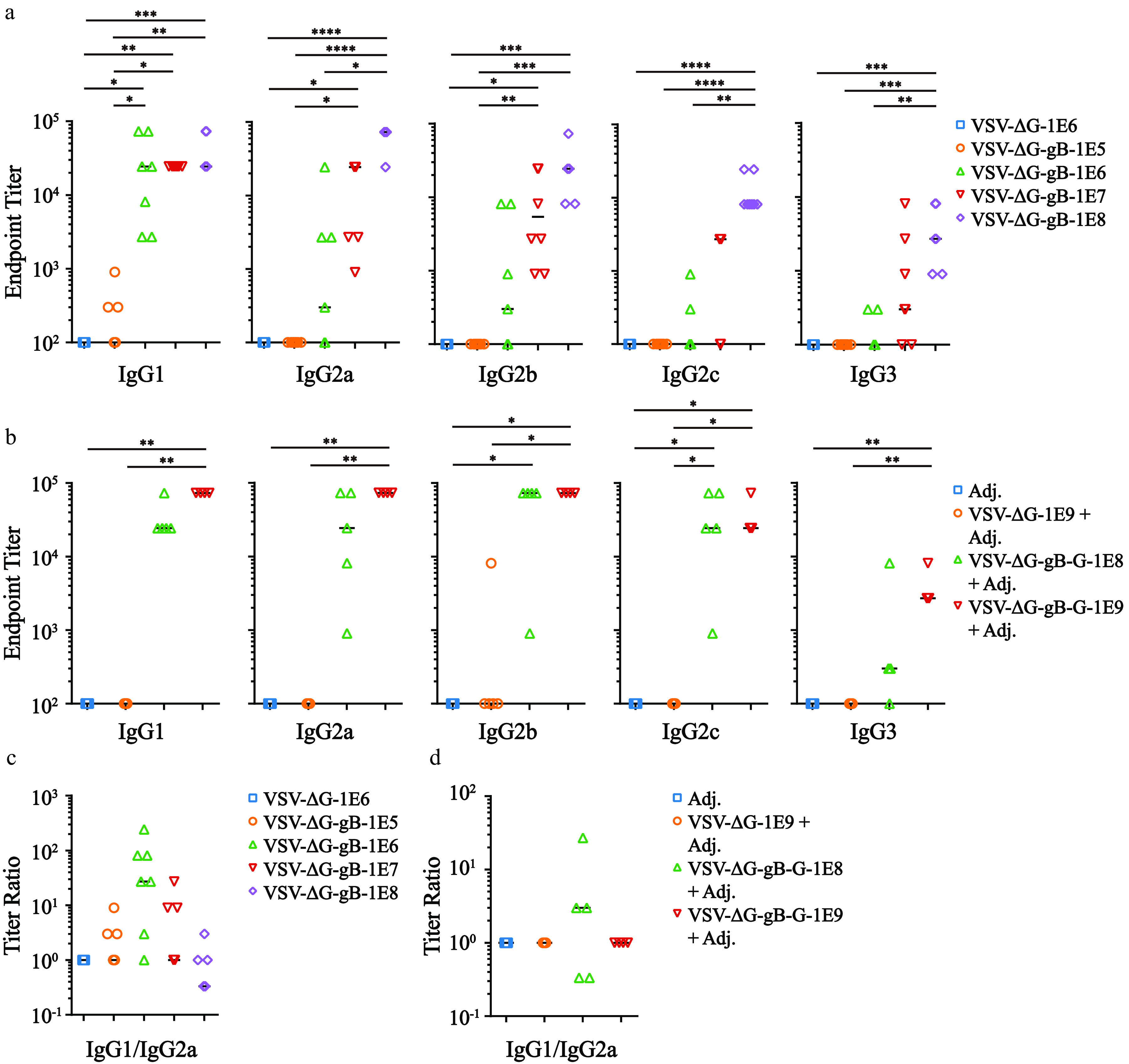
Profiling characteristics of the immune response of VSV-ΔG-gB or VSV-ΔG-gB-G vaccination by IgG subtype titers. (a and b) The endpoint titers of the indicated IgG subtype immunized with VSV-ΔG-gB and VSV-ΔG-gB-G. Each dot represents the outcome of individual animals, and each horizontal bar represents the median for each within each group. The Kruskal–Wallis test following Dunn’s multiple-comparison test was adopted to perform statistical analysis among groups. *, *P* < 0.05; **, *P* < 0.01; ***, *P* < 0.001; ****, *P* < 0.0001. (c and d) The ratio of IgG1 to IgG2a immunized with VSV-ΔG-gB and VSV-ΔG-gB-G. Each dot represents the outcome of individual animals, and each horizontal bar represents the median of each within each group.

To obtain a closer look at the immune characteristics, we applied ELISA to detect five IgG subtypes, IgG1, IgG2a, IgG2b, IgG2c, and IgG3, and endpoint titers of the 2nd boost serum immunized with either VSV-ΔG-gB (Fig. 4a) or VSV-ΔG-gB-G (Fig. 4b). Immune responses of treatment groups in every IgG subtype were significantly higher than those in the negative-control groups. Like the trend of total IgG, IgG subtype titers increased as dosage increased. In every IgG subtype, the homogeneity also increased as the dosage increased, stressing the benefit of high-dosage vaccinations. Most interestingly, unlike previously reported gp350 vaccines (14, 16), the ratio of IgG1 to IgG2a approached one as the dosage increased (Fig. 4c). Even with the humoral-prone aluminum adjuvant, the trend was the same (Fig. 4d). As a lower ratio of IgG1 to IgG2a reflects the propensity of cellular responses (47), VSV-ΔG-gB or VSV-ΔG-gB-G might induce stronger cellular responses than gp350-centered vaccines (14, 16). It remains unknown whether this property is shared by other carriers presenting EBV gB (21, 30, 31) or is the unique benefit of VSV as a vaccine carrier.

### VSV-ΔG-gHgL preferentially elicited a high ratio of epithelial nAbs.

**FIG 5:**
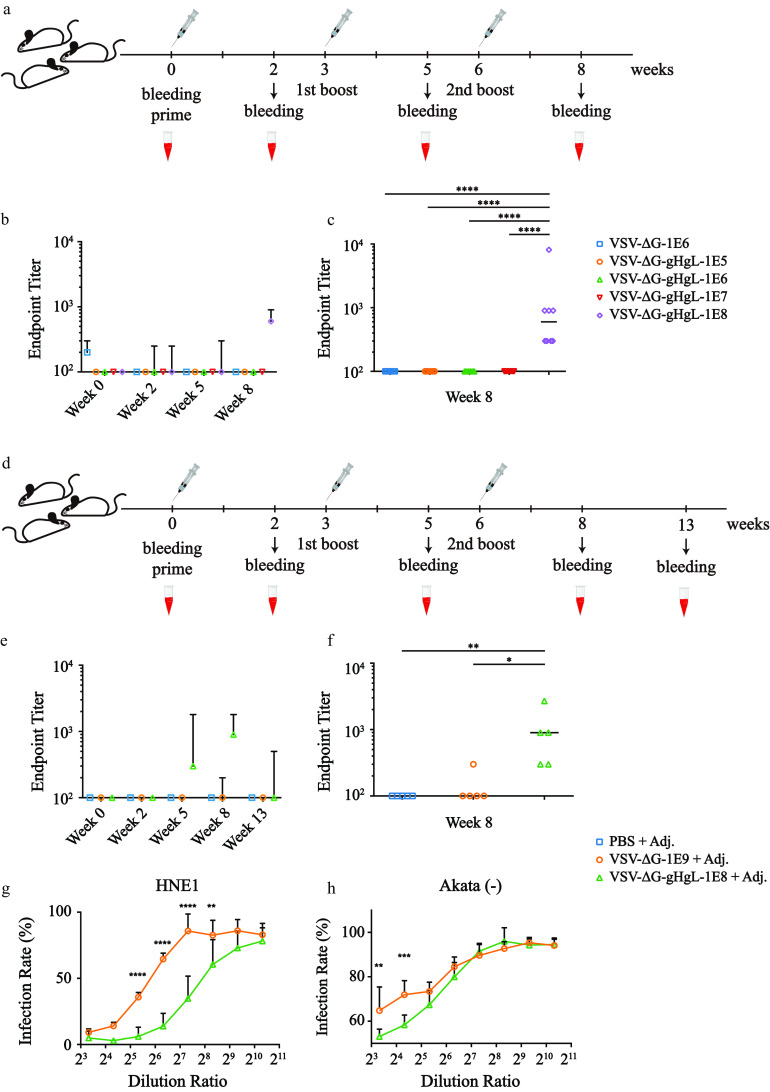
High ratio of epithelial-biased neutralizing antibodies elicited by VSV-ΔG-gHgL vaccination. (a) The immunization schedule diagram without adjuvant. (b) The kinetics of IgG endpoint titer of the *in vivo* experiments immunized with VSV-ΔG-gHgL without adjuvant. Each dot represents the median titer within each group, and each error bar denotes the interquartile range within each group. *n* = 8 mice per group. All animals were in good status throughout the experiment. (c) IgG endpoint titers of the 2nd boost were extracted from panel b. The Kruskal–Wallis test following Dunn’s multiple-comparison test was adopted to perform the statistical analysis. (d) The immunization schedule diagram with adjuvant. (e) The kinetics of IgG endpoint titer of the *in vivo* experiments immunized with VSV-ΔG-gHgL with aluminum adjuvant. Each dot represents the median titer within each group, and each error bar denotes the interquartile range within each group. *n* = 5 mice per group. All animals were in good status throughout the experiment. (f) IgG endpoint titers of the 2nd boost were extracted from panel e. The Kruskal–Wallis test following Dunn’s multiple-comparison test was adopted to perform the statistical analysis. (g and h) The neutralizing results with the 2nd boost serum vaccinated with VSV-ΔG-gHgL with aluminum adjuvant. Infection rates were calculated by dividing the raw infection rates by the mean raw infection rates of three positive controls where no serum was mixed with Akata-EBV prior to EBV infection. Each dot represents the mean normalized infection rate of the indicated dilution ratio, and the error bar denotes the standard deviation. Two-way ANOVA following Sidak’s multiple-comparison test was adopted to perform statistical analysis. *, *P* < 0.05; **, *P* < 0.01; ***, *P* < 0.001; ****, *P* < 0.0001.

Unlike trimeric EBV gB, which is similar to VSV-G, the presentation of monomeric EBV gHgL was far more difficult, where only 94.8 ng of gHgL was detected by the double-antibody sandwich ELISA in 1E9 qTiter virions (Fig. 1i). Vaccination without adjuvant and bleeding schedule of VSV-ΔG-gHgL was basically the same as that of VSV-ΔG-gB (Fig. 5a). The kinetics of total IgG reflected moderate but detectable humoral responses immunized with 1E9 qTiter VSV-ΔG-gHgL (Fig. 5b). Compared to the negative control and lower dosage groups, IgG responses in the higher dosage group were significantly higher (Fig. 5c). Due to moderate humoral responses, we carried out another independent *in vivo* experiment with aluminum adjuvant (Fig. 5d). The kinetics of total IgG showed incremental immune responses to triple vaccinations (Fig. 5e), and the total IgG of VSV-ΔG-gHgL was significantly higher than that of the negative controls (Fig. 5f). Thirteen weeks postprime, antibodies against gHgL dropped (Fig. 5e). Only one out of five mice in the VSV-ΔG-gHgL-1E8 + Adj. group processed detectable antibodies. The duration difference between gB- and gHgL-presenting groups may predominantly be attributed to the antibody titer at the 2nd boost.

To identify nAbs, we conducted neutralizing experiments. Strikingly, with moderate total IgG titers, the relative infection rates were significantly different in both epithelial cells and B cells between the vaccinated group and the control group. Moreover, the endpoint titers of epithelial cells reached 1:320, while B cells reached merely 1:20 (Fig. 5f and g). Taken together, VSV-ΔG-gHgL preferentially elicited a high ratio of neutralizing antibodies and biased epithelial neutralization, which was similar to previously reported EBV gHgL nanoparticle vaccines (32).

### An additive effect of combination vaccination of VSV-ΔG-gB-G and VSV-ΔG-gHgL.

**FIG 6:**
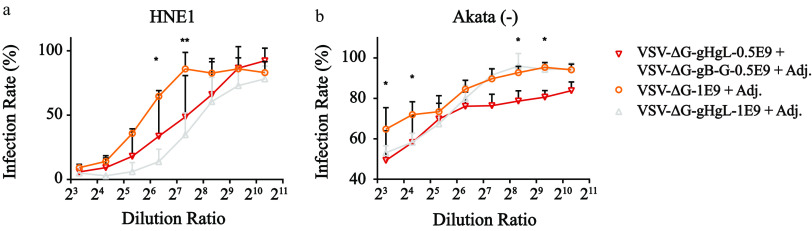
Additive effects with the combination vaccination of VSV-ΔG-gHgL and VSV-ΔG-gB-G. (a and b) The neutralizing results of epithelial cells and B cells overlaid with the VSV-ΔG-gHgL-alone vaccination results. By implementation of VSV-ΔG-gB-G, the setback of VSV-ΔG-gHgL vaccination inducing weak B cell nAbs was rescued, indicating the unique value of combinatorial vaccination. Infection rates were calculated by dividing the raw infection rates by the mean raw infection rates of three positive controls where no serum was mixed with Akata-EBV prior to EBV infection. Each dot represents the mean normalized infection rate of the indicated dilution ratio, and the error bar denotes the standard deviation. *n* = 5 mice per group. All animals were in good status throughout the experiment. Statistical analysis adopted two-way ANOVA following Sidak’s multiple-comparison test. *, *P* < 0.05; **, *P* < 0.01; ***, *P* < 0.001; ****, *P* < 0.0001.

Given that bias nAbs were elicited by VSV-ΔG-gB-G and VSV-ΔG-gHgL, we performed a combinatorial vaccination by immunizing both 0.5E9 qTiter VSV-ΔG-gB-G and 0.5E9 qTiter VSV-ΔG-gHgL simultaneously. Epithelial cell neutralizing experiments showed no significant titer difference compared to the VSV-ΔG-gHgL-only vaccination group (Fig. 6a). However, B cell neutralizing effects were significantly boosted by VSV-ΔG-gB administration (Fig. 6b), indicating an additive effect of the combination vaccination.

## DISCUSSION

Prophylactic vaccines against human tumor viruses, such as HBV and HPV, could significantly reduce their associated liver cancer and cervical cancer (48, 49). Given a stunning over 90% infection rate of EBV and, in 2010, 142,979 cancer deaths attributable to EBV (50, 51), a prophylactic vaccine that prevents EBV infection in both epithelial cells and B cells should greatly reduce the incidence of those diseases. Various vaccine design strategies are available (52). In this study, we pioneered VSV-based EBV vaccines presenting EBV gB or gHgL. Systemic *in vivo* experiments confirmed that as little as 13 ng of soluble gB-equivalent VSV-ΔG-gB could elicit a 1E3 IgG EC_50_ titer against gB. Vaccination of VSV-ΔG-gB-G elicited nAbs for epithelial cells and B cells. With as little as 9.48 ng of soluble gHgL equivalent to VSV-ΔG-gHgL, 1E3 IgG endpoint titers against gHgL and epithelial and B cell nAbs could be detected. The results suggested that VSV is a supreme EBV vaccine carrier that plays a key adjuvant-like role in vaccination, and with more in-depth studies, VSV-based EBV vaccines are strong candidates for clinical trials.

By using EBV gHgL nanoparticle vaccines, Bu et al. showed that higher titers of epithelial nAbs than B cell nAbs could be detected (10^4.9^ to 10^3.0^ 50% inhibitory concentrations [IC_50_]) (32). In previous studies utilizing oligomers, Cui et al. showed that both EBV gB and EBV gHgL elicited a higher ratio of epithelial nAbs than B cell nAbs (∼10^2.5^ to ∼10^2^ IC_50_) (31). Perez et al. also conducted neutralization experiments using serum from EBV gB- or EBV gHgL-centered but latent protein combined vaccines, where instead of serum dilution, they measured the neutralizing effects by EBV dilution (21), so it is difficult to extract comparable neutralizing titer information from the study. Nonetheless, we revealed that nAbs elicited by VSV-ΔG-gB-G vaccination preferentially prevented B cell infection (>1,024 to 40 endpoint titer) (Fig. 3a and b), while nAbs elicited by VSV-ΔG-gHgL preferentially prevented epithelial cell infection (320 to 20 endpoint titer) (Fig. 5g and h), which had not been observed. We presumed that these might be attributed to the significant difference in the presented dominant epitopes, which could be further explored by applying cryo-electron microscopy (cryo-EM) structural analysis.

As an important criterion for Th1/Th2 polarization, IgG1/IgG2a implies a propensity for humoral and cellular immune responses, where a lower ratio indicates stronger Th1 responses and, thus, a cellular immune response (47). Currently, there are only two studies out of all EBV gp350-, gB-, and gHgL-related vaccine developments presenting the IgG1/IgG2a ratio in which the animals were immunized by gp350-centered vaccines (14, 16). Unlike the high IgG1/IgG2a ratios shown in these two studies, the IgG1/IgG2a ratio of the nonadjuvant VSV-ΔG-gB or the adjuvant VSV-ΔG-gB-G vaccination was approximately 1. No further cellular immune response study on vaccines centered at EBV gp350, gB, and gHgL was reported, which might result from the neglect of the importance of cellular immunity in prophylactic vaccine development (53). In a more general context, in-depth studies on T cell immune responses of these glycoproteins are rare (54), partly due to the characteristics of the EBV life cycle, where the expression levels of gp350, gB, and gHgL are low or absent in latency (1). However, with advances in immunology, it is worth paying more attention to the role of cellular immunity in EBV prophylactic vaccine development, especially T cell responses against gB and gHgL presented on the surface of VSV.

Many benign and malignant diseases were etiologically associated with EBV infection (55–59). One potential drawback of the designed vaccines described here was the requirement of normal immune responses. That is to say, for those primary immunodeficiency-related or acquired immunodeficiency-related severe EBV infection, which might induce various lymphomas, vaccination is of little help (56, 57). On the other hand, antigens presented on the surface of VSV are categorized as lytic proteins, which means that these proteins will not produce during EBV latency. Once EBV establishes latent infection in host cells, antibodies against gB or gHgL might not help recognize these abnormal cells. T cell immunity plays a vital role in controlling the latent infection (56, 57). For example, the defect of T cells in cognate or acquired immunodeficiency leads to EBV-associated lymphoma (56, 57). Moreover, currently there is no defined characterization of viremia before the onset or during the development of EBV-associated carcinoma, and the effects of antibodies elicited by vaccination after latent infection is established require in-depth study.

Thus, we proposed that vaccination with EBV late protein-centered prophylactic vaccines such as gB, gHgL, or gp350 should reach maximum effects when administered prior to primary EBV infection; however, vaccination in a high-risk NPC population might serve as a secondary option where EBV might reactivate and nasopharyngeal epithelial cell infection might not be established. Lastly, due to the high compatibility of VSV vector, we have already initiated studies on VSV-based EBV therapeutic vaccines where latent proteins were displayed on the surface of VSV. In the future we will be able to directly compare EBV controlling efficacy with late or latent protein-centered vaccines and help to identify the optimal immune program to eliminate EBV infection and its associated diseases.

Taken together, we reported the first VSV-based EBV prophylactic vaccines. With long-tested vector safety, potent antigenicity, and distinct immune response characteristics, these vaccine constructs unfolded new possibilities of EBV vaccine research and development. To pave the road to clinical studies and simplification of virion production and increase of surface presentation density by integration of EBV gB or EBV gHgL genes into VSV-ΔG genome, cross-species antigenicity and safety evaluation, structural studies on dominant epitopes, and in-depth cellular immune response mechanisms are required and will be elaborated in future studies.

## MATERIALS AND METHODS

### Cells, plasmids, and antibodies.

BSR-T7, BHK, and 293T cells were grown in Dulbecco’s modified Eagle medium containing high glucose (DMEM; Gibco) supplemented with 10% fetal bovine serum (FBS). HNE1 and Akata(−) cells were grown in RPMI 1640 with 10% FBS. 293F cells were suspended and cultured in serum-free medium (UP1000; Union). All cells were cultured at 37°C with 5% CO_2_.

Plasmids pBS-VSVFL, pBS-VSV-N, pBS-VSV-P, and pBS-VSV-L were provided by Mingzhou Chen’s lab. Plasmid pBS-VSVΔG was generated from pBS-VSVFL, which carries the full-length genome of VSV, by replacing the gene sequence of the VSV G protein with GFP. pCAGGS-VSV-G, pCAGGS-EBV-gH, pCAGGS-EBV-gL, and pCAGGS-EBV-gB, carrying the full-length VSV-G, EBV-gH, EBV-gL, and EBV-gB genes, were constructed according to the manufacturer’s instructions.

Mouse anti-VSV-G monoclonal antibody (MAb) IE9F9 and anti-VSV-M MAb 23H12 were purchased from KeraFast. Goat anti-rabbit and goat anti-mouse polyclonal antibodies were purchased from Thermo Fisher. Antibodies against EBV gB and gHgL are described below.

### Recovery of recombinant virus.

The protocol for the generation of recombinant VSV was modified from Whitt (42). BSR-T7 cells were seeded in 10-cm dishes at a density of 80% overnight. The cells were then infected with vTF7-3 at a multiplicity of infection (MOI) of 1 for 1 h before being transfected with the pBS-VSV-N (5 μg), pBS-VSV-P (3 μg), pBS-VSV-L (1 μg), pCAGGS-VSV-G (8 μg), and pBS-VSV-△G genomes (5 μg) through Lipofectamine 3000 (Invitrogen). The transfection medium was discarded and replaced with 10% FBS DMEM. Forty-eight hours later, the cells were frozen and thawed 3 times, and the clarified supernatant was collected by centrifugation. vTF7-3 was removed by passing through 0.22-μm filters twice. A fresh BHK cell monolayer was prepared to be transfected with pCAGGS-VSV-G and then infected with the recombinant virus stock for amplification. A single clone of recombinant VSV-ΔG-VSV-G was picked up during titration in an agar plate, followed by amplification in BHK cells.

VSV-ΔG and VSV-ΔG-gB were generated by transfecting 293T cells with plasmid pCAGGS or pCAGGS-EBV-gB (pCAGGS-EBV-gH and pCAGGS-EBV-gL for VSV-ΔG-gHgL) and infecting them with VSV-ΔG-VSV-G 24 h after transfection at an MOI of 5 for 1 h. Cells were washed twice with phosphate-buffered saline (PBS) and incubated in 5% FBS DMEM for 24 h. Supernatant was harvested through 0.22-μm filters. Virions were isolated from the supernatant by pelleting through a 20% sucrose cushion for 2 h and resuspended in PBS. Virions were then further purified and concentrated by 20% to 50% sucrose density gradient ultracentrifugation at 40,000 × *g* for 15 h. The target layer was extracted and pelleted through a 20% sucrose cushion at 100,000 × *g* for 2 h. Virions were resuspended in PBS and stored at −80°C.

### Negative-stain EM imaging.

A 5-μL aliquot of purified samples was applied to a glow-discharged grid with a continuous carbon layer (Electron Microscopy China) for 1.5 min. The grid was blotted by filter paper to absorb the excrescent samples. The grid was then stained with 8 μL uranyl acetate for 40 s. Redundant liquid was absorbed using filter paper. The grid was transferred to a Talos 120 C transmission electron microscope (Thermo Fisher Scientific) performed at 120 kV in low-dose mode and imaged with a Ceta 16 M CMOS detector (Thermo Fisher Scientific). Data collection was operated at ×57,000 magnification.

### LC-MS.

The presentation of EBV gHgL on VSV was detected by mass spectrometry. Purified virions of VSV-ΔG and VSV-ΔG-gHgL were lysed by a 5× sample buffer, boiled at 100°C for 10 min, and resolved by SDS-PAGE. To reduce contamination, electrophoresis was stopped as soon as the samples ran down into the separation gel, and the gel was resected and digested, followed by liquid chromatography and mass spectrometry. Data analysis was carried out with ProteomeDiscovery 2.5.

### Protein expression and purification.

The DNA sequence encoding EBV gL (residues 24 to 137; UniProt ID G3CKR4) linked to EBV gH (residues 19 to 678; UniProt ID G3CKS5; C-terminal 6× His tag) with a (G4S)3 linker and the DNA sequence encoding EBV gB (residues 25 to ∼683, UniProt ID R4R670) were cloned into pcDNA3.1. A CD5 signal peptide DNA sequence was inserted into the N terminus of genes of interest. Antibodies against EBV gHgL, E1D1 (45), and AMMO1 (60) and recombinant gHgL and gB were expressed in 293F cells. The supernatant was collected and purified by Ni^+^ affinity chromatography (Ni Sepharose Excel; Cytiva). For gHgL, the eluate was concentrated and purified by size exclusion chromatography (SEC) (Superdex 200 Increase 10/300 GL; Cytiva) running at 50 mM NaCl and 50 mM HEPES, pH 7.5; fractions were collected and applied to anion-exchange chromatography (HiTrap Capto Q; 5 mL; Cytiva) and eluted by linear ion-strength increment; fractions were concentrated and purified by SEC (Superdex 200 Increase 10/300 GL) running at 50 mM HEPES and 150 mM NaCl, pH 7.5. For gB, the eluate was concentrated and purified by SEC (Superdex 200 Increase 10/300 GL; Cytiva) running at 50 mM NaCl and 150 mM HEPES, pH 7.5. For E1D1 and AMMO1, the supernatant was captured by protein A beads (L00210; GenScript) followed by SEC (Superdex 200 Increase 10/300 GL; Cytiva) running in PBS.

### SDS-PAGE and immunoblotting.

Purified virions were lysed with a sample buffer for 10 min on ice, boiled with bromophenol blue at 100°C for 10 min, and resolved via 10% SDS-PAGE. The gel was either stained with Coomassie brilliant blue or transferred to a polyvinylidene difluoride (PVDF) membrane for immunoblot analysis. For immunoblot analysis, the membrane was blocked with 5% milk in phosphate-buffered saline (PBS) with 0.1% Tween 20 (PBST) for 1 h before being incubated with primary antibodies overnight and then incubated with secondary antibodies for another 1 h.

### Mouse vaccination.

BALB/c or C57 female mice were purchased from Guanddong Medical Laboratory Animal Center or GemPharmatech. Mice were divided into different groups as indicated and immunized subcutaneously (s.c.) at the age of 6 or 8 weeks. For vaccination with adjuvant, an aluminum adjuvant (Imject alum adjuvant; Invitrogen) and virions were mixed at a ratio of 1:1 before vaccination. For primary, 1st boost, and 2nd boost vaccination, the same batch of vaccines was injected at week 0, week 3, and week 6. Blood was collected at week 0, week 2, week 5, and week 8.

All animal experiments were conducted in accordance with the regulations of the Institutional Animal Care and Use Committee, Sun Yat-sen University Cancer Center, Sun Yat-sen University, and the protocol was approved by the committee. All animals were cared for humanely in accordance with the requirements of the committee.

### ELISA.

For ELISA, we coated the high binding 96-well plates (number 42592; Corning) with 100 ng purified soluble antigens in 100 µL PBS and allowed them to complete binding by incubating plates overnight at 4°C. The buffer block was prepared by dissolving bovine serum albumin (BSA) in PBST (1% Tween 20, unless otherwise specified) to a final concentration of 5% (wt/vol). We discarded the supernatant and add 350 µL buffer block to each well. We allowed complete blocking by incubating plates at 37°C for 1 h. We discarded the supernatant and washed plates with PBST. We prepared a 3-fold serum dilution series starting at a ratio of 1:100 with buffer block. We incubated plates at 37°C for 1 h. We discarded the supernatant and washed plates with PBST. Total IgG, IgG1, IgG2a, IgG2c, IgG2b, IgG2c, IgG3, IgA, IgE, and IgM were detected by horseradish peroxidase (HRP)-conjugated antibodies (ab6789, ab97240, ab97245, ab97250, ab97255, ab97260, ab97235, ab99574, and ab97230; Abcam) diluted at a ratio of 1:10,000 with buffer block. We incubated plates at 37°C for 1 h. We discarded the supernatant and washed plates with PBST. We added 100 µL TMB substrate per well. The reaction was stopped by adding 100 µL of 1:12 hydrochloride acid to distilled water per well. We read plates at the optical density at 450 nm (OD_450_) and OD_630_. For subsequent data analysis, Δ(OD_450_, OD_630_) was used as the input data.

### Double-antibody sandwich ELISA.

The double-antibody sandwich ELISA proceeded similarly to regular ELISA, with the following modifications. First, 100 ng gB antibody 3A5 (unpublished data) or 100 ng gHgL antibody 1D8 (61) was coated. In addition to the tested virions, purified EBV gB ectodomain or EBV gHgL ectodomain was diluted 3-fold. The amount of presented gB or gHgL of the capture virions was detected by the other binding site nonoverlapping gB antibody, 3A3-HRP (unpublished data), or gHgL antibody AMMO1-HRP (60). We extracted the log linear region as the working dynamic test range. We fit the linear curve and then calculated the soluble equivalent EBV gB or gHgL on the surface of VSV-ΔG-gB, VSV-ΔG-gB-G, or VSV-ΔG-gHgL.

### Neutralizing assessment.

Akata-EBV, which is GFP positive, was prepared using the Akata cell line as previously described (27, 29, 46). Five thousand HNE1 cells were seeded in each well of 96-well plates. We allowed cells to grow overnight. We prepared a 2-fold serum dilution series with 10% FBS DMEM containing antibiotics. We added an equal amount of Akata EBV to each well. We allowed serum binding to virus for 1 h at 37°C with 5% CO_2_. We discarded medium of HNE1 and replaced it with the serum-virus mix. We allowed virus infection for 2 h at 37°C with 5% CO_2_. We replaced it with fresh 10% FBS DMEM containing antibiotics. Twenty-four hours later, the cells were digested with trypsin, and GFP signal was detected by flow cytometry (CytoFLEX LX; Beckman).

For B cell line neutralization evaluation, 10,000 Akata(−) cells in 50 μL FBS-containing medium were seeded in each well of 96-well plates. We prepared 100 μL serum-virus mix/well as previously described. We allowed continuous virus infection for 24 h. GFP signal was detected by flow cytometry (CytoFLEX LX; Beckman).
